# When Social Interaction Backfires: Frequent Social Interaction During the COVID-19 Pandemic Is Associated With Decreased Well-Being and Higher Panic Buying

**DOI:** 10.3389/fpsyg.2021.668272

**Published:** 2021-07-29

**Authors:** Hyunji Kim, Arnd Florack

**Affiliations:** Faculty of Psychology, University of Vienna, Vienna, Austria

**Keywords:** social connectedness, social interaction, COVID-19, well-being, general confidence level, panic buying

## Abstract

The present research investigated a backfiring effect of social interaction on well-being and general confidence in Western populations during the COVID-19 pandemic. Across two studies, we observed that stronger self-other connectedness and frequent social communication with others during the first few weeks into the quarantine period were associated with worsened well-being and decreased general confidence. In Study 1 (*n* = 331), we showed that people who reported higher social connectedness and more frequent social interaction experienced declined well-being. In Study 2 (*n* = 327), we replicated the backfiring effect and showed that those who engaged in frequent social interaction, especially in COVID-19 related conversations, reported decreased general confidence, which mediated the accelerating effect of social interaction on panic buying. Overall, our findings indicated that frequent social interaction under a highly novel and uncertain crisis can relate to negative consequences on mental health and behavior.

## Introduction

The COVID-19 outbreak which started in Wuhan, the capital of Hubei Province in the People’s Republic of China has led to over seven million infected cases and 400,000 deaths as of early June 2020 across the globe (Worldometers, 2020). During this time, to slow down the spread of COVID-19, few essential government measures had been implemented. Of those measures, rules for staying in quarantine and keeping the distance, so called the practice of “social distancing” were amongst crucial measures to be imposed for effectively flattening the curve of daily confirmed cases (Hamzelou, 2020; Piguillem and Shi, 2020)^1^. This measure, however, has been reported to produce various negative psychological consequences that are related to well-being (e.g., Ingram et al., 2020; Wei, 2020) and compliance behavior (Brooks et al., 2020).

Based on accumulated research on the stress-relieving role of social support and interaction (e.g., Thoits, 1995; Cohen, 2004; Ye et al., 2020), one effective strategy to counter the negative psychological consequences of social distancing would be to engage in active social communications to strengthen social bonds. However, in a pandemic situation, whether engaging in social interactions, specifically, actively engaging in communications with others, foster intended outcomes might be an open question. Although social support from significant others and interpersonal communications have been reported to alleviate negative psychological reactions toward health crises (Griffin and Dunwoody, 2000; Mak et al., 2009; Wang et al., 2014), sudden quarantine rules and unknown global challenges with much uncertainty might lead to negative social interactions backfiring the expected function of social interaction. In fact, negative social interactions led by circumstantial restrictions such as failing to provide emotional or instrumental help, invading another’s privacy, or depriving of confidence or hope have been largely ignored in major social support and health research (Lincoln, 2000). Hence, our goal was to examine the extent to which social connectedness and social interaction affected well-being and negative consequences (e.g., panic buying) during the COVID-19 pandemic across Western countries. Our data collection took place on the 19th of April (Study 1), and the 1st of May (Study 2) in 2020.

In challenging times, increased stress can lower the ability to cope with and adjust to the difficult situation due to the depletion of psychological or physical resources, which in turn, can lead to worsened mental and physical health (Dohrenwend and Dohrenwend, 1974; Lazarus and Folkman, 1984; Pearlin, 1989; Brown and Harris, 2012). Previous research has continuously shown that the strength of social connection and perceived availability of social support act as psychological resources to combat detrimental emotional and behavioral consequences of negative situations (Lazarus, 1966; Pearlin and Schooler, 1978; Billings and Moos, 1981; Lazarus and Folkman, 1984; Cohen et al., 1985). According to the stress-buffer hypothesis (Cohen, 2004), the process of such buffering effect occurs through re-appraisal and re-interpretation of the adverse events in a way that social relationships and support buffer the psychological impact of stressors for those undergoing challenging times (House, 1981; Cohen and Wills, 1985; Cohen, 1988).

In case of the COVID-19 outbreak, besides the acute psychological reactions (e.g., fear, anxiety), the outbreak has also generated a cascade of long-lasting impacts on occupational (e.g., job loss, increased risk for essential workers) and social life in general, creating multiple stressors. The societal and individual damages the outbreak has produced continued to be unresolved without specific remedies for a substantial amount of time. The absence of solutions adds further harm to coping with the situation and to maintaining the psychological well-being (Turner and Avison, 1992). Based on a bulk of social support literatures, keeping close social relationships and engaging in active social interaction with significant others might be a cure for alleviating negative psychological consequences because through social communications, one should be able to reappraise the pandemic situation to lessen the negative aspect of the event and restore hope.

Nevertheless, previous research rarely looked at a pandemic situation wherein weekly new measures were announced based on somewhat ambiguous and highly versatile information. In fact, the types of social interaction during the first few weeks into the quarantine period might have inclined to confirming uncertainty and magnifying fear rather than successfully reappraising the pandemic situation. According to the social amplification of risk framework (Kasperson et al., 1988; Kasperson, 2014) and the concept of informational social influence (Cantril, 1952), a risk event or hazard can be amplified by various individual and social tools for exchanging information. Such ways of social communication, so called the word-of mouth, can easily be accelerated because people are highly motivated by social goals such as emotional regulation and information acquisition (Berger, 2014). Such accelerated communication can lead to physical harm (Burns et al., 1993), reactions such as blame and dread (Wirz et al., 2018) and society impacts such as political attention by public officials, loss of sales, and increased costs due to regulations (Renn et al., 1992) as consequences. Therefore, in an effort to understand the COVID-19 situation, those who have engaged in active social interaction might have amplified the negative aspects of COVID-19 leading to increased negative psychological consequences. To test this, we examined whether stronger social connectedness and active social interaction during this time led to such a backfiring effect.

Another relevant social construct in rapidly changing situations involving extreme uncertainty and risk is trust. Trust in society plays an important role in coping with the unknown situation (Siegrist and Cvetkovich, 2000) and novel societal risks against uncertainty and threat (Keller et al., 2011), as a psychological basis of social relations for strengthening group membership and shared values. Built on trust, people develop a certain level of confidence that the given situation will improve (Siegrist et al., 2005). Collective trust has been known as a vital social capital for people to overcome feelings of uncertainty and alleviate negative consequences of risk perception, especially in the absence of knowledge (Luhmann, 1989; Earle and Cvetkovich, 1995). In order to cope with lack of knowledge and high uncertainty, people often rely on trust to reduce the complexity of the unknown situation (Siegrist and Cvetkovich, 2000). Although the construct of trust in a pandemic situation can be distributed across multiple referents (i.e., technological, political and societal institutions, and toward other people in general), we focused on the overarching belief in the system and society as a whole, namely general confidence (Luhmann, 1988, 1989). While trust is built toward generalized individuals or groups to be relied on (Rotter, 1967), general confidence is built toward generalized objects or systems emphasizing certainty and control rather than intentions and values (Earle and Siegrist, 2006; Earle et al., 2007). As the COVID-19 government measures relate to general trust in the societal system, reflecting a general belief that the society will persevere and strive through the challenge, we concluded to focus on whether the backfiring effect of active social interactions also transferred to lowering the general confidence level.

When the level of general confidence decreases, one predictable behavioral consequence in crises is panic buying (e.g., Arafat et al., 2020). Due to lack of psychological buffers to cope with societal threat, one might engage in behaviors that can boost self-preservation (Clarke, 2002; Min et al., 2020; Oosterhoff and Palmer, 2020). Despite the display of altruism and prosocial behavior prevalent in crises (Drury, 2018), panic buying at supermarkets and drugstores has been a widespread response to the COVID-19 outbreak. We argue that one reason for this behavior might be due to threatened general confidence resulting from exchanging views about how dramatic the situation is. Thus, we tested whether the weakened general confidence level via social communication would be associated with more panic buying.

In sum, our hypotheses were as follows. We hypothesized that strong social connectedness and frequent social interaction will be associated with worsened well-being and increased stress due to the amplifying effect of social influence on risk perception.

H1a: Stronger social connectedness and more frequent social interaction predict worsened well-being and increased stress.

We also hypothesized that the risk amplifying effect (frequent social interaction) on well-being and general confidence is mainly due to social communications about COVID-19 related topics.

H1b: Social interaction but mainly the communications about COVID-19 related topics will be associated with worsened well-being and decreased general confidence level.

Lastly, we hypothesized that frequency of social communication about COVID-19 related topics would predict panic buying and this relation will be mediated by the decreased general confidence level.

H2: Decreased general confidence level mediates the effect of frequent social communication (about COVID-19 related topics) on higher panic buying.

We tested H1a in Study 1 and H1b and H2 in Study 2. All studies were ethically approved and conducted in accordance with the guidelines and regulations by the Institutional Review Board at the department of Occupational, Economic, and Social Psychology at University of Vienna. Participants were paid 6 pounds (British sterling) an hour rate in Study 1 and 6.3 pounds (British sterling) an hour rate in Study 2 in their own currencies.

## Study 1

In Study 1, we investigated the moderating role of social connectedness and social interaction during the pandemic on changes in self-reported stress and well-being before and after the COVID-19 outbreak. Before testing our predictions, we operationalized social connectedness as a trait measure for closeness of social relationships with others in general. In order to gauge social closeness, we focused on measuring the tendency for interdependence and inclusion of others to the self. Accordingly, we combined two well-known measures assessing the construct of social closeness: level of interdependent self-construal (Singelis, 1994) and the self-other inclusion scale (Aron et al., 1992). We also operationalized the term social interaction as engaging in social conversations with significant others mainly via online tools during the pandemic. Thus, we gauged the frequency of social interaction by measuring the frequency of online social interaction, general inquiry of status (i.e., how someone is doing) with family, friends, and colleagues. Our additional measure for the government rule compliance indicated that our participants followed the quarantine rules and kept social distance from anyone except those living with them after the outbreak (see Supplementary Materials).

### Method

#### Participants

Before conducting analyses, 36 participants were excluded due to failing our attention check items (i.e., Please choose “strongly disagree”). 331 participants (59.2% Male; *M*_age_ = 26.95, *SD*_age_ = 8.91) recruited via a widely used online platform (Prolific.co) were entered the analyses. Prior to recruitment, our sample size was calculated via G^∗^Power to detect a relatively small effect (*f*^2^ = 0.04) with over 80% power. We calculated the sample size using a total number of 6 predictors (social connectedness, social interaction, information search, age, gender, education) while having 2 tested predictors (social connectedness, social interaction) in a linear multiple regression analysis for testing the R^2^ increase. The sample size we needed was 244. Given that our study was the first to explore the detrimental effect of social connectedness and social interaction on well-being, and given that the potential drop-out rate was unknown, we increased our sample size to ensure enough power. Participants were provided with an online informed consent form and gave consent by clicking the continue button to proceed to the survey.

#### Measures

Participants were instructed to fill out a questionnaire given the measures below. In order to control for any method bias, we chose the independent self-construal measure as a marker variable (see Supplementary Materials).

##### Social Connectedness

Based on our operationalization of social connectedness (i.e., interdependence and social inclusion), we used two well-known scales to gauge social connectedness in our study. First, we used the 10 item self-construal measure whereby 5 items measured interdependent self-construal (e.g., “I will sacrifice myself-interest for the benefit of the group I am in,” “My relationships are more important than my own accomplishments”) and another 5 items measured independent self-construal (e.g., “I do my own thing, regardless of what others think”, “I’d rather say no directly than risk being misunderstood”; Singelis, 1994; D’Amico and Scrima, 2016). Second, we used the inclusion of others in the self (IOS) scale (Aron et al., 1992) to measure the extent to which a conceptual overlap occurs between the self and other. We combined the interdependent self-construal and the IOS scale as a composite score for social connectedness by standardizing the mean values of the two scales and averaging them into one composite variable (see Supplementary Materials for CFA of the composite variable).

##### Social Interaction

Participants indicated to what extent they agreed with two statements after the outbreak, “I have actively engaged in online interaction via SNSs and chat apps with friends and family members for social interaction,” and “I have actively engaged in finding out how other people (friends and family, colleagues, etc.) are doing compared to how I am doing,” on a seven-point scale (1 = *strongly disagree*, 7 = *strongly agree*).

##### Information Search

To gauge information search, participants filled out two items measuring frequency of information search. Participants reported how often they engaged in information search for the COVID-19 and information sharing about the COVID-19 on a seven-point scale (1 = *never*, 7 = *every time*).

##### Stress *Before* and *After* the Outbreak

Participants indicated how much stress they were under *before* and *after* the COVID-19 outbreak, each using a single item stress measure (Watson, 1988), on a five-point scale (1 = *felt very slightly or not at all*, 5 = *felt very much*). Change in stress was calculated by subtracting the stress measure *before* the outbreak from the stress measure *after* the outbreak. Higher values indicated worsened stress.

##### Well-Being *Before* and *After* the Outbreak

We used the Scale of Positive and Negative Experience (SPANE) developed by Diener et al. (2010), a widely used measure for gauging subjective well-being (e.g., Huppert and So, 2013; Söllner et al., 2021). SPANE contained six items to assess positive feelings (e.g., positive, joyful, sad) and six items to assess negative feelings (e.g., unpleasant, sad, afraid). Participants indicated how much emotion they felt *before* and *after* the COVID-19 outbreak, on a five-point scale each (1 = *very rarely or never*, 5 = *very often or always*). Change in well-being was calculated so that higher values always indicated worsened well-being (i.e., less positive and more negative feelings).

### Results

#### Demographics and Descriptive Statistics

Our valid sample size for Study 1 was 331 (59.2% Male; *M*_age_ = 27.98, *SD*_age_ = 0.05). Participants reported their education level given 4 options: (1). Did not finish high school (4.2%), (2). High school graduation (39.9%), (3). College graduation (37.8%), postgraduate graduation (18.1%). Participants also reported their nationality given an open text box: 16.9% Polish, 16% Portuguese, 14.5% British, 4.5% American, 4.2% Greek, 3.9% Canadian and the rest were mainly European nationals. 299 participants (90.3%) reported that their country of residence was the same as their nationality.

Overall, participants reported that they felt more stressed (*before*: *M* = 2.65, *SD* = 1.16, *after*: *M* = 3.11, *SD* = 1.21), *t*(330) = −6.38, *p* < 0.001, felt less positive affect (*before*: *M* = 3.54, *SD* = 0.66, *after*: *M* = 3.17, *SD* = 0.69), *t*(330) = 11.28, *p* < 0.001, and felt more negative affect (*before*: *M* = 2.58, *SD* = 0.73, *after*: *M* = 2.87, *SD* = 0.76), *t*(330) =, *p* < 0.001, *after* compared to *before* the COVID-19 outbreak.

#### Intercorrelations of All Measures

Intercorrelational results showed that social connectedness positively correlated with the decrease in positive affect, *r*(329) = 0.18, *p* = 0.001, the increase in negative affect, *r*(329) = 0.20, *p* < 0.001, and the stress increase, *r*(329) = 0.16 *p* = 0.004. Frequency of social interaction also highly correlated with the decrease in positive affect, *r*(329) = 0.24, *p* < 0.001, the increase in negative affect, *r*(329) = 0.17 *p* = 0.002, for negative affect), and stress increase, *r*(329) = 0.17, *p* = 0.002, whereas frequency of information search did not (all *r*s < 0.098, all *p*s > 0.07; see Table 1).

**TABLE 1:** Intercorrelations for Measures Included in Study 1 (*n* = 331).

Measures	*M* (*SD*)	1.	2.	3.	4.	5.	6.	7.	8.	9.
1. Social connectedness	0.00 (0.80)	(0.56)								
2. Independent SC	4.41 (1.09)	–0.05	(0.68)							
3. Social interaction	4.95 (1.53)	0.34**	–0.10	(0.66)						
4. Information search	4.13 (1.31)	0.17**	0.04	0.35**	(0.65)					
5. Δ stress	0.10 (1.20)	0.16**	0.04	0.17**	0.10	–				
6. Δ positive	0.38 (0.61)	0.18**	0.04	0.24**	0.11*	0.52**	(0.89,0.88)			
7. Δ negative	0.29 (0.65)	0.20**	0.04	0.16**	0.08	0.50**	0.64**	(0.84,0.84)		
8. Age	26.95 (8.91)	−0.15**	0.16*	–0.10	0.02	0.01	–0.05	0.001	–	
9. Education	2.70 (0.81)	–0.04	0.03	0.04	0.02	0.06	0.09	0.07	0.29**	–

SC, Self-Construal. Cronbach’s alpha is provided in parentheses where necessary (two values indicate *before* and *after* the COVID-19 measures). Changes are coded so that higher values indicate worsened well-being and stress.

*p < 0.05, **p < 0.01.

#### Moderated Multiple Regression

**FIGURE 1:**
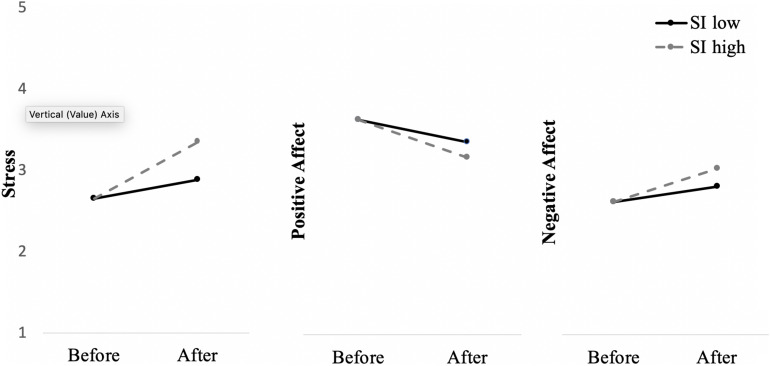
Moderation effects of social interaction on stress and well-being (positive and negative affects) *before* and *after* the outbreak in Study 1. SI, Social Interaction; SI low and high indicate mean values −1 SD and + 1 SD, respectively.

We performed a multiple moderation analysis for repeated measures (MEMORE; Montoya, 2019) on each outcome variable, regressing social connectedness and social interaction onto changes in stress and well-being between *before* and *after* the outbreak. Our analyses revealed that social connectedness and social interaction significantly contributed to the increase in stress, *F*(2, 328) = 7.01, *p* = 0.001, *R*^2^ = 0.04 (social connectedness: β = 0.12, *se* = 0.10; social interaction: β = 0.13, *se* = 0.05), decrease in positive affect, *F*(2, 328) = 12.20, *p* < 0.001, *R*^2^ = 0.07 (social connectedness: β = 0.11, *se* = 0.04; social interaction: β =. 20, *se* = 0.02), and increase in negative affect, *F*(2, 328) = 8.97, *p* < 0.001. *R*^2^ = 0.05 (social connectedness: β = 0.16, *se* = 0.05; social interaction: β = 0.12, *se* = 0.02), indicating that those who engaged in more frequent and active social interaction reported stronger decrease in well-being and higher stress (see Figure 1 and Table 2).

**TABLE 2:** Moderated Regression Analyses in Study 1 and Study 2.

	Decrease of positive affect				Increase of negative affect				Stress increase			
Predictors	*b* (*se*)	95% CI for *b*	β	*sr* ^2^	*b* (*se*)	95% CI for *b*	β	*sr* ^2^	*b* (*se*)	95% CI for *b*	β	*sr* ^2^
**Study 1 (*n* = 331)**												
Social connectedness	0.09 (0.04)	0.001, 0.169	0.11*	0.11	0.13 (0.05)	0.036, 0.217	0.16**	0.15	0.19 (0.10)	0.003, 0.378	0.12*	0.11
Social interaction	0.08 (0.02)	0.036, 0.125	0.20**	0.19	0.05 (0.02)	0.004, 0.099	0.12*	0.11	0.12 (0.05)	0.017, 0.214	0.13*	0.13

	**Decrease of positive affect**				**Increase of negative affect**				**General confidence decrease**			
**Predictors**	***b* (*se*)**	**95% CI for *b***	**β**	***sr*^2^**	***b* (*se*)**	**95% CI for *b***	**β**	***sr*^2^**	***b* (*se*)**	**95% CI for *b***	**β**	***sr*^2^**

**Study 2 (*n* = 327)**			(*R*^2^ = 0.05***)				(*R*^2^ = 0.07***)				(*R*^2^ = 0.03**)	
*Step 1*												
Social connectedness	0.13 (0.05)	0.036, 0.216	0.15**	0.15	0.17 (0.05)	0.078, 0.268	0.20***	0.19	0.0004 (0.06)	−0.112, 0.113	0.0004	0.0004
Social interaction	0.07 (0.03)	0.021,0.124	0.15**	0.15	0.07 (0.03)	0.014, 0.122	0.14*	0.13	0.10 (0.03)	0.037, 0.166	0.17**	0.17
		(*R*^2^ = 0.10***, Δ*R*^2^ = 0.04***)				(*R*^2^ = 0.13***, Δ*R*^2^ = 0.12***)				(*R*^2^ = 0.08***, Δ*R*^2^ = 05***)		
*Step 2*												
Social connectedness	0.10 (0.05)	0.006, 0.185	0.12*	0.11	0.13 (0.05)	0.041, 0.228	0.15**	0.15	−0.04 (0.06)	−0.152, 0.070	−0.04	−0.04
Social interaction	0.04 (0.03)	−0.01, 0.096	0.09	0.09	0.03 (0.03)	−0.024, 0.085	0.06	0.06	0.06 (0.03)	−0.004, 0.126	0.11	0.10
COVID-19 conversation	0.14 (0.03)	0.067, 0.202	0.22**	0.21	0.17 (0.04)	0.103, 0.244	0.27***	0.25	0.18 (0.04)	0.100, 0.268	0.25***	0.23

***p < 0.001, **p < 0.01, *p < 0.05.

**sr*^2^ indicates semi-partial (part) correlational coefficient. Changes in positive affect, negative affect, stress, and general confidence are coded so that higher values indicated decreased well-being, increased stress, and decreased general confidence.*

In addition, to control for any potential retrospective biases that might have occurred in the *before* measures, we have conducted a hierarchical regression model. Our analyses revealed that the effects of our predictor variables (social connectedness and social interaction) on the *after* measures (positive affect, negative affect, and stress) were significant after accounting for the *before* measures (see Supplementary Table 3-1).

### Discussion

Our findings showed that well-being and stress were worsened for those who reported higher social connectedness and social interaction. Unlike popular believes and empirical evidence from social support literatures, the feeling of connectedness with other people and staying socially close to others surprisingly backfired exerting a detrimental effect on mental health during the first few weeks of the quarantine period. According to our rationale, situations like the COVID-19 outbreak are unique in that reappraisals might not be effective and instead, amplification of a risk event through social influence might occur. Indeed, our findings showed that social communications after the outbreak have increased negative psychological consequences.

Following the results observed in Study 1, we examined whether the content of social interaction, especially conversations about COVID-19 related topics uniquely contributed to the backfiring effect on subjective well-being. Furthermore, we investigated whether the frequency of engaging in conversations about COVID-19 related topics contributed to increased distrust in society (i.e., general confidence). We also examined the moderating role of social interaction and COVID-19 related conversations on changes in well-being and general confidence level. Finally, we examined whether COVID-19 conversations during the outbreak boosted panic buying through the decreased level of general confidence.

## Study 2

In Study 2, we hypothesized that social connectedness, frequent social interaction but mainly the conversations about COVID-19 related topics would predict decreased well-being and decreased level of general confidence. We expected that the changes in well-being and general confidence level would be moderated by risk relevant social interaction namely, COVID-19 conversation. We also hypothesized that frequent COVID-19 related conversations would be associated with higher panic buying and the relation between COVID-19 conversation and panic buying would be mediated by the decreased level of general confidence. Additionally, to gauge more specific attitudes and emotions related to the pandemic situation, we included measures for uncertainty, anxiety and fear as exploratory variables.

### Method

#### Participants

Before conducting analyses, 28 participants were excluded due to failing our attention check items. 327 participants (53.2% Male; *M*_age_ = 26.94, *SD*_age_ = 9.02) were recruited via Prolific were entered the analyses. Prior to recruitment, our sample size was calculated via G^∗^Power to detect a relatively small effect (*f*^2^ = 0.04) with over 80% power. We calculated the sample size using a total number of 7 predictors (social connectedness, social interaction, COVID-19 conversation, information search, age, gender, education) while having 3 tested predictors (social connectedness, social interaction, COVID-19 conversation) in a linear multiple regression analysis for testing the R^2^ increase. The sample size we needed was 277. Given that the new variable we introduced in Study 2 is novel, we increased our sample size to ensure enough power. Participants were provided with an online informed consent form and gave consent by clicking the continue button to proceed to the survey. Participants who took part in Study 1 were not eligible to participate in Study 2.

#### Measures

Participants were instructed to fill out a questionnaire including social connectedness, social interaction, and information search, and changes in subjective well-being measures used in Study 1 and additionally, the following measures below.

##### COVID-19 Conversation

To gauge the extent to which people talked about COVID-19 related topics when engaging in social interactions, participants indicated across five items, how often they engaged in conversations on each type of the topics, (1). COVID-19 news or reports on media, (2). Public reaction to COVID-19 (e.g., rallies, panic buying, donation, etc.), (3). Personal risk of getting infected with COVID-19, (4). Overall uncertainty about the COVID-19 situation, (5). Influence of COVID-19 on normal life style, given a seven-point scale (1 = *never*, 7 = *every time*).

##### Uncertainty, Anxiety, and Fear *Before* and *After* the Outbreak

Adapted from the stress measure used in Study 1, participants indicated how uncertain, anxious, and fearful they felt *before* and *after* the COVID-19 outbreak, each given a six-point scale (1 = *not at all*, 6 = *extremely*). Changes in uncertainty, anxiety, and fear were calculated so that higher values indicated increased negative feelings.

##### General Confidence Level *Before* and *After* the Outbreak

To measure the general confidence level, we used the 6-item general confidence scale developed by Keller et al. (2011). Example items are “Our society is well equipped to solve future problems,” “The future safety and security of our population is assured.” Participants indicated to what extend they agreed with each statement given a 6-point scale (1 = strongly disagree, 6 = strongly agree) *before* and *after* the COVID-19 outbreak. The change variable was calculated so that higher values indicated decreased general confidence.

##### Panic Buying

Due to absence of the existing measure at the time of data collection, four author-generated items assessed panic buying behavior. Participants reported how true each statement was given a seven-point scale (1 = *very untrue of me*, 7 = *very true of me*). The items were “I worried that certain products (e.g., toilet papers, pasta, hand soaps, etc.) at supermarkets would run out,” “I bought household supplies (e.g., toilet papers, detergent) and/or certain groceries (e.g., pasta, rice, canned food, frozen food) a little more than usual,” “I bought household supplies (e.g., toilet papers, detergent) and/or certain groceries (e.g., pasta, rice, canned food, frozen food) a little earlier than usual,” “My shopping behavior did not change at all (reversed item).”

### Results

#### Demographics and Descriptive Statistics

Our valid sample size for Study 2 was 327 (53.2% Male; *M*_age_ = 26.94, *SD*_age_ = 9.02). Participants reported their education level given 4 options: (1). Did not finish high school (4.9%), (2). High school graduation (42.5%), (3). College graduation (39.4%), postgraduate graduation (13.1%). Participants also reported their nationality given an open text box: 21.4% Polish, 18.6% British, 16.5% Portuguese, 8.8% Italian, 3% American, and the rest were mainly European nationals. 297 participants (90.8%) reported that their country of residence was the same as their nationality.

Overall, the level of negative affect (*before*: *M* = 2.66, *SD* = 0.69; *after*: *M* = 3.08, *SD* = 0.74), uncertainty (*before*: *M* = 3.17, *SD* = 1.23; *after*: *M* = 4.23, *SD* = 1.33), anxiety (*before*: *M* = 3.08, *SD* = 1.40; *after*: *M* = 4.06, *SD* = 1.42), and fear (*before*: *M* = 2.59, *SD* = 1.22; *after*: *M* = 3.71, *SD* = 1.32), significantly increased *after*, compared to *before*, the COVID-19 outbreak (all *t*s > −0.12, all *p*s < 0.001). The level of positive affect (*before*: *M* = 3.62, *SD* = 0.65; *after*: *M* = 3.06, *SD* = 0.71) and the general confidence level (*before*: *M* = 3.61, *SD* = 1.04; *after*: *M* = 2.77, *SD* = 1.05), significantly decreased *after*, compared to *before*, the COVID-19 outbreak (all *t*s > 15, all *p*s < 0.001).

#### Intercorrelations of All Measures

Intercorrelational results showed that social connectedness, social interaction, and COVID-19 conversation highly correlated with changes in well-being (all *r*s > 0.17 all *p*s < 0.01), and social interaction and COVID-19 conversation also highly correlated with increased uncertainty, fear, and anxiety (all *r*s > 0.18, all *p*s < 0.01; see Table 3). We point out that low internal consistencies of the interdependent self-construal measure we found in both studies (Study 1:0.56, Study 2:0.62) should be given caution. Previous studies have also reported relatively low internal consistencies gauging interdependent self-construal (e.g., alphas = 0.63,0.64; Rohmann et al., 2012; Besta, 2018) alarming researchers for conducting similar future studies using such a measure. Despite the low Cronbach’s alphas, the interdependent self-construal highly correlated with the inter-personal closeness measure in our design (Study 1: *r* = 0.3 Study 2: *r* = 0.32) to form a composite social connectedness variable. Lastly, our main effects for Study 1 and Study 2 were identical when using the inter-personal closeness measure alone for indicating social connectedness.

**TABLE 3:** Intercorrelations for Measures Included in Study 2 (*n* = 327).

Measures	*M* (*SD*)	1.	2.	3.	4.	5.	6.	7.	8.	9.	10.	11.	12.	13.
1. Social connectedness	0.00 (0.81)	(0.62)												
2. Independent SC	4.39 (1.08)	–0.02	(0.70)											
3. Information search	4.04 (1.26)	0.08	0.01	(0.56)										
4. Social interaction	5.06 (1.42)	0.19**	0.10	0.36**	(0.71)									
5. COVID-19 conversation	4.42 (1.11)	0.22**	–0.06	0.49**	0.31**	(0.81)								
6. Panic buying	3.80 (1.46)	0.02	–0.09	0.19**	0.17**	0.26**	(0.81)							
7. Δpositive	0.56 (0.68)	0.18**	0.003	0.09	0.18**	0.27**	0.10	(0.88,0.90)						
8. Δnegative	0.43 (0.72)	0.22**	0.04	0.21**	0.17**	0.32**	0.15*	0.75**	(0.84,0.85)					
9. Δ general confidence	0.81 (0.83)	0.05	–0.04	0.07	0.18**	0.26**	0.18**	0.33**	0.30**	(0.83,0.82)				
10. Δ uncertainty	1.05 (1.39)	0.06	–0.09	0.19**	0.23**	0.32**	0.18**	0.34**	0.36**	0.45**	–			
11. Δ fear	1.13 (1.37)	0.13*	0.005	0.21**	0.27**	0.32**	0.19**	0.41**	0.48**	0.43**	0.61**	–		
12. Δ anxiety	0.98 (1.42)	0.17**	–0.03	0.19**	0.18**	0.27**	0.21**	0.41**	0.47**	0.35**	0.60**	0.67**	–	
13. Age	26.94 (9.02)	–0.01	0.07	–0.02	0.13*	0.07	0.08	0.13*	0.14*	0.12*	0.12*	0.18**	0.16**	
14. Education	2.61 (0.78)	0.002	–0.03	–0.01	0.16*	0.11	0.14*	0.01	0.07	0.12*	0.16**	0.11*	0.11*	0.28**

SC, Self-Construal. Cronbach’s alpha is provided in parentheses where necessary (two values indicate *before* and *after* the COVID-19 measures). Changes are coded so that higher values indicate worsened well-being, lowered general confidence, increased uncertainty, fear and anxiety.

*p < 0.05, **p < 0.01.

#### Moderated Multiple Regression and Mediation

To test unique contributions of social connectedness, social interaction and COVID-19 conversation to changes in well-being and general confidence, we performed multiple moderation analyses for repeated measures (MEMORE; Montoya, 2019). The regression analyses revealed significant predictions for the decrease of positive affect *F*(3, 323) = 11.63, *p* < 0.001, *R*^2^ = 0.10 (social connectedness: β = 0.12, *se* = 0.05; social interaction: β = 0.09, *se* = 0.03, COVID-19 conversation: β = 0.22, *se* = 0.03), the increase of negative affect, *F*(3, 323) = 16.04, *p* < 0.001, *R*^2^ = 0.13 (social connectedness: β = 0.15, *se* = 0.05; social interaction: β = 0.06, *se* = 0.03, COVID-19 conversation: β = 0.27, *se* = 0.04), and the decrease of general confidence, *F*(3, 323) = 9.69, *p* < 0.001, *R*^2^ = 0.08 (social connectedness: β = −0.04, *se* = 0.06; social interaction: β = 0.10, *se* = 0.03, COVID-19 conversation: β = 0.25, *se* = 0.04). As seen in Table 2, our analyses revealed that social connectedness and COVID-19 conversation significantly predicted changes in well-being but only COVID-19 conversation predicted changes in general confidence over and above social connectedness and social interaction.

In addition, to control for any potential retrospective biases that might have occurred in the *before* measures, we have conducted a hierarchical regression model. Our analyses revealed that the effects of our predictor variables (social connectedness, social interaction, and COVID-19 conversation) on the *after* measures (positive affect, negative affect, and general confidence) were significant after accounting for the *before* measures (see Supplementary Table 3-2).

**FIGURE 2:**
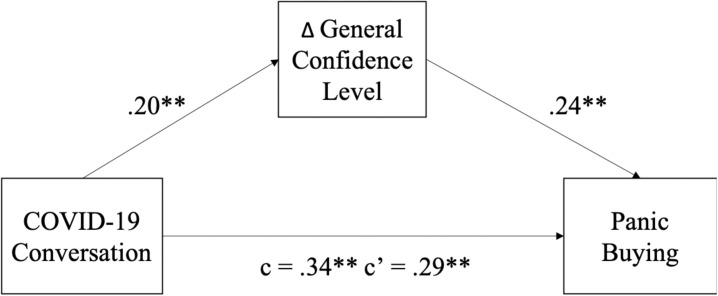
Mediation effect of Δ general confidence level between COVID-19 conversation and panic buying observed in Study 2. ^∗∗^p < 0.01.

Lastly, we tested the mediating role of the general confidence level on the relation between COVID-19 conversation and panic buying. Bootstrapped mediation analyses (10,000 resamples) revealed that the decreased general confidence level partially mediated the effect of COVID-19 conversation on panic buying [indirect effect = 0.04, 95% bias corrected and accelerated confidence interval (BCa CI) = 0.002,0.089; total effect = 0.34, 95% BCa CI = 0.202,0.480; direct effect = 0.30 95% CI = 0.156,0.442], indicating that one explanation for frequent COVID-19 conversations resulting in more panic buying was through a decreased level of general confidence (see Figure 2).

### Discussion

Our results in Study 2 confirmed the backfiring effect of social interaction, especially that of COVID-19 related conversations, on worsened well-being and decreased general confidence. Those who reported more frequent and active social interactions felt less positive affect, more negative affect, and reported lower general confidence after the COVID-19 outbreak. More frequent COVID-19 conversations were associated with higher panic buying through the decreased level of general confidence.

Importantly, our regression analyses showed that when including COVID-19 conversation as an additional moderator, the effect of social interaction on well-being disappeared. In other words, our results indicated that the backfiring effect of social interaction observed in our studies might be mainly due to engaging in social communications on COVID-19 related topics. Overall, our findings suggest that daily social interaction about the risk event might have contributed to worsened well-being and decreased trust in society, which in turn might have led to panic buying.

Because of the low reliability of the interdependent self-construal measure, future studies might examine whether formative measurement models (Bollen and Diamantopoulos, 2017) which assess interdependent self-construal in different domains are more adequate than reflective measurement models. For example, a formative measurement model has previously been used to develop a person-group fit measure (Li et al., 2019) which is conceptually similar to interdependent self-construal. Another approach would be to examine the predictive validity of response time measures of self-representations which reveal the degree to which the self is prioritized over the social others (Kim and Florack, 2021).

## General Discussion

Across two studies, we demonstrated that stronger social connectedness and frequent social interaction during the first few weeks into the COVID-19 quarantine period consistently contributed to decreased well-being, increased stress, and decreased general confidence. As hypothesized, the drop observed in general confidence mediated the accelerating effect of COVID-19 conversation on panic buying. Overall, our findings indicate that social communication in this specific pandemic period amplified the negative psychological consequences and lowered the general trust level in society, which in turn partially contributed to a maladaptive behavioral response.

A bulk of social support literature shows that in difficult times social bonds and social interactions play a crucial role as a stress-buffer via reappraisals (Cohen, 2004). However, social interactions can also turn into a negative viral reaction via a word of mouth type of communications which amplify risk perception of the negative event. Our findings are in line with the social amplification of risk framework (Kasperson et al., 1988) that social influence can act as an amplifier for the risk event to spiral into negative psychological and behavioral consequences.

Nevertheless, our findings do not undermine the potential positive effect of social interaction as a stress-buffer. In Study 2, the essential component of social interaction that ultimately contributed to the backfiring effect was conversations about COVID-19 related topics. Given the novelty of the COVID-19 situation around the globe, exchanging uncertain and partial information about COVID-19 might have led people to symbolize the situation in a negative way and to confirm the given circumstances as facing a global catastrophe. However, other components of social interactions that are not directly measured in our studies might have contributed to stress reliving and well-being preserving effects. For instance, felt availability of social support or felt belongingness might have played a buffering role against negative consequences (e.g., Hou et al., 2020).

One limitation of the present study is that our findings do not draw a direct causal relation between social interaction and well-being. One could argue a reverse direction that people might have interacted with each other more frequently because they felt worse. Although our findings do not completely rule out this possibility, the fact that our individual difference measure for social connectedness consistently predicted worsened well-being (see Table 2) indicates that close social relation must have preceded changes in well-being. This pattern observed in our study also indicates that the effects of social connectedness and social interaction might have undergone separate mechanisms influencing well-being. While people who reported higher social connectedness might have suffered from social isolation, people who reported higher engagement of social communication might have been influenced by the social amplification. However, we acknowledge that this relation could be bidirectional in nature, in a way that close social relation and active social interaction can be associated with more negative consequences which in turn can boost the motivation and longing for more social interactions until a satisfying resolution occurs.

Another limitation is that, even though we demonstrated that the covariates measured in the studies (i.e., age, gender, education, nationality) do not hinder our conclusions (see Supplementary Tables 2, 6), other covariates that are not measured in our studies might have influenced the results. For instance, living conditions (e.g., living alone, with family, or in a shared flat) and marital status might have affected the well-being and stress during the quarantine period. However, recent studies conducted during the COVID-19 pandemic reported that subjective loneliness but not living alone was associated with mental health (Cabello et al., 2021) and living alone did not necessarily harm well-being for older adults (Fingerman et al., 2021), unless diagnosed with dementia or mild cognitive impairment (Hashimoto et al., 2020).

Our findings highlight that despite the positive effect of strong social connection during negative events, social interaction under extremely uncertain and sudden social changes such as the COVID-19 pandemic can also lead to unexpected consequences in well-being. Given that our testing period corresponds to the beginning of the implementation of quarantine rules, the public reactions toward the new restrictions might have been intensified. This particular period, due to higher motivation for emotional regulation and information acquisition, might have led to a more negative spiral of social communication. At the individual level, being wary of the potential harm that engaging in conversations about the pandemic situation itself might be detrimental to well-being is important to guide one’s social interactions in a more desirable way. Our findings also imply that at the beginning of implementing such government measures, providing clear information and instructions might be utmost essential for avoiding such negative effects of social influence on mental health and societal trust.

## Data Availability Statement

The datasets presented in this study can be found in online repositories. The names of the repository/repositories and accession number(s) can be found below: https://osf.io/xjmwh/.

## Ethics Statement

The studies involving human participants were reviewed and approved by the Institutional Review Board of the Department of Occupational, Economic, and Social Psychology at University of Vienna. The patients/participants provided their written informed consent to participate in this study.

## Author Contributions

HK and AF designed the study, interpreted the results, wrote the original manuscript, and revised it together. HK collected and analyzed the data. AF supervised the data collection and analyses. Both authors contributed to the article and approved the submitted version.

## Conflict of Interest

The authors declare that the research was conducted in the absence of any commercial or financial relationships that could be construed as a potential conflict of interest.

## Publisher’s Note

All claims expressed in this article are solely those of the authors and do not necessarily represent those of their affiliated organizations, or those of the publisher, the editors and the reviewers. Any product that may be evaluated in this article, or claim that may be made by its manufacturer, is not guaranteed or endorsed by the publisher.

## Footnotes

1 See https://www.acaps.org/covid-19-government-measures-dataset (accessed May 2020).

## References

[B1] Arafat S. Y. Kar S. K. Marthoenis M. Sharma P. Apu E. H. Kabir R. (2020). Psychological underpinning of panic buying during pandemic (COVID-19). *Psychiatry Res.* 289:113061. 10.1016/j.psychres.2020.113061 PMC7202808 32413711

[B2] Aron A. Aron E. N. Smollan D. (1992). Inclusion of other in the self scale and the structure of interpersonal closeness. *J. Pers. Soc. Psychol.* 63 596–612. 10.1037/0022-3514.63.4.596

[B3] Berger J. (2014). Word of mouth and interpersonal communication: a review and directions for future research. *J. Consum. Psychol.* 24 586–607. 10.1016/j.jcps.2014.05.002

[B4] Besta T. (2018). Independent and interdependent? Agentic and communal? self-construals of people fused with a group. *Ann. Psychol.* 34 123–134. 10.6018/analesps.34.1.266201

[B5] Billings A. G. Moos R. H. (1981). The role of coping responses and social resources in attenuating the stress of life events. *J. Behav. Med.* 4 139–157. 10.1007/BF00844267 7321033

[B6] Bollen K. A. Diamantopoulos A. (2017). In defense of causal-formative indicators: a minority report. *Psychol. Methods* 22 581–596. 10.1037/met0000056 26390170 PMC6670294

[B7] Brooks S. K. Webster R. K. Smith L. E. Woodland L. Wessely S. Greenberg N. (2020). The psychological impact of quarantine and how to reduce it: rapid review of the evidence. *Lancet* 395 912–920. 10.1016/s0140-6736(20)30460-8 32112714 PMC7158942

[B8] Brown G. W. Harris T. (2012). *Social Origins of Depression: a Study of Psychiatric Disorder in Women.* New York: Routledge.

[B9] Burns W. J. Slovic P. Kasperson R. E. Kasperson J. X. Renn O. Emani S. (1993). Incorporating structural models into research on the social amplification of risk: implications for theory construction and decision making. *Risk Analysis* 13 611–623. 10.1111/j.1539-6924.1993.tb01323.x

[B10] Cabello M. Izquierdo A. Leal I. (2021). Loneliness and not living alone is what impacted on the healthcare professional’s mental health during the COVID-19 outbreak in Spain. *Health Soc. Care Commun.* 10.1111/hsc.13260 Online ahead of print. 33761161 PMC8250561

[B11] Cantril H. (1952). *The Invasion from Mars: a Study in the Psychology of Panic.* New Jersey: Princeton.

[B12] Clarke L. (2002). Panic: myth or reality? *Contexts* 1 21–26. 10.1525/ctx.2002.1.3.21 33021500

[B13] Cohen S. (1988). Psychosocial models of social support in the etiology of physical disease. *Health Psychol.* 7 269–297. 10.1037/0278-6133.7.3.269 3289916

[B14] Cohen S. (2004). Social relationships and health. *Am. Psychol.* 59 676–684. 15554821 10.1037/0003-066X.59.8.676

[B15] Cohen S. Mermelstein R. Kamarck T. Hoberman H. M. (1985). “Measuring the functional components of social support,” in *Social Support: Theory, Research and Applications*, ed. Sarason I. G. (Berlin: Springer), 73–94. 10.1007/978-94-009-5115-0_5

[B16] Cohen S. Wills T. A. (1985). Stress, social support, and the buffering hypothesis. *Psychol. Bull.* 98 310–357 10.1037/0033-2909.98.2.310 3901065

[B17] D’Amico A. Scrima F. (2016). The Italian validation of Singelis’s Self-Construal Scale (SCS): a short 10-item version shows improved psychometric properties. *Curr. Psychol.* 35 159–168. 10.1007/s12144-015-9378-y

[B18] Diener E. Wirtz D. Tov W. Kim-Prieto C. Choi D. W. Oishi S. (2010). New well-being measures: short scales to assess flourishing and positive and negative feelings. *Soc. Indic. Res.* 97 143–156. 10.1007/s11205-009-9493-y

[B19] Dohrenwend B. S. Dohrenwend B. P. (1974). *Stressful Life Events: Their Nature and Effects.* Michigan: Wiley.

[B20] Drury J. (2018). The role of social identity processes in mass emergency behaviour: an integrative review. *Eur. Rev. Soc. Psychol.* 29 38–81. 10.1080/10463283.2018.1471948

[B21] Earle T. C. Cvetkovich G. (1995). *Social Trust: Toward a Cosmopolitan Society.* Westport, CT: Praeger.

[B22] Earle T. C. Siegrist M. (2006). Morality information, performance information, and the distinction between trust and confidence. *J. Appl. Soc. Psychol.* 36 383–416. 10.1111/j.0021-9029.2006.00012.x

[B23] Earle T. C. Siegrist M. Gutscher H. (2007). “Trust, risk perception, and the TCC model of cooperation,” in *Trust in Cooperative Risk Management: Uncertainty and Scepticism in the Public Mind*, eds Siegrist M. Earle T. C. Gutscher H. (London: Earthcscan), 144–163.

[B24] Fingerman K. L. Ng Y. T. Zhang S. Britt K. Colera G. Birditt K. S. (2021). Living alone during COVID-19: social contact and emotional well-being among older adults. *J. Gerontol. Ser. B* 76 e116–e121. 10.1093/geronb/gbaa200 PMC7717423 33196815

[B25] Griffin R. J. Dunwoody S. (2000). The relation of communication to risk judgment and preventive behavior related to lead in tap water. *Health Commun.* 12 81–107. 10.1207/S15327027HC1201_05 10938908

[B26] Hamzelou (2020). World in lockdown. *New Scientist* 245:3275. 10.1016/S0262-4079(20)30611-4 PMC7270163 32518458

[B27] Hashimoto M. Suzuki M. Hotta M. Nagase A. Yamamoto Y. Hirakawa N. (2020). The influence of the COVID-19 outbreak on the lifestyle of older patients with dementia or mild cognitive impairment who live alone. *Front. Psychiatry* 11:570580. 10.3389/fpsyt.2020.570580 PMC7661779 33192695

[B28] Hou J. Yu Q. Lan X. (2020). COVID-19 infection risk and depressive symptoms among young adults during quarantine: the moderating role of grit and social support. *Front. Psychol.* 11:577942. 10.3389/fpsyg.2020.577942 PMC7820677 33488448

[B29] House J. S. (1981). *Work Stress and Social Support. Reading.* Boston, MA: Addison-Wesley.

[B30] Huppert F. A. So T. T. (2013). Flourishing across Europe: application of a new conceptual framework for defining well-being. *Soc. Indic. Res.* 110 837–861. 10.1007/s11205-011-9966-7 23329863 PMC3545194

[B31] Ingram J. Maciejewski G. Hand C. J. (2020). Changes in diet, sleep, and physical activity are associated with differences in negative mood during COVID-19 lockdown. *Front. Psychol.* 11:2328. 10.3389/fpsyg.2020.588604 PMC7492645 32982903

[B32] Kasperson R. (2014). Four questions for risk communication. *J. Risk Res.* 17 1233–1239. 10.1080/13669877.2014.900207

[B33] Kasperson R. E. Renn O. Slovic P. Brown H. S. Emel J. Goble R. (1988). The social amplification of risk: a conceptual framework. *Risk Anal.* 8 177–187.

[B34] Keller C. Siegrist M. Earle T. C. Gutscher H. (2011). The general confidence scale: coping with environmental uncertainty and threat. *J. Appl. Soc. Psychol.* 41 2200–2229. 10.1111/j.1559-1816.2011.00811.x

[B35] Kim H. Florack A. (2021). Immediate self-information is prioritized over expanded self-information across temporal, social, spatial, and probability domains. *Q. J. Exp. Psychol.* 10.1177/17470218211004208 Online ahead of print. 33719761 PMC8358571

[B36] Lazarus R. S. (1966). *Psychological Stress and the Coping Process*. New York, NY: McGraw–Hill.

[B37] Lazarus R. S. Folkman S. (1984). *Stress, Appraisal, and Coping.* Berlin: Springer.

[B38] Li C. S. Kristof-Brown A. L. Nielsen J. D. (2019). Fitting in a group: theoretical development and validation of the multidimensional perceived person-group fit scale. *Pers. Psychol.* 72 139–171. 10.1111/peps.12295

[B39] Lincoln K. D. (2000). Social support, negative social interactions, and psychological well-being. *Soc. Serv. Rev.* 74 231–252. 10.1086/514478 26594064 PMC4651456

[B40] Luhmann N. (1988). “Familiarity, confidence, trust: problems and alternatives,” in *Trust: Making and Breaking Cooperative Relations*, ed. Diego G. (Oxford: Blackwell), 94–107.

[B41] Luhmann N. (1989). *Vertrauen: Ein Mechanismus der Reduktion Sozialer Komplexität.* Brunei: Universiti Teknologi Brunei

[B42] Mak W. W. Law R. W. Woo J. Cheung F. M. Lee D. (2009). Social support and psychological adjustment to SARS: the mediating role of self-care self-efficacy. *Psychol. Health* 24 161–174. 10.1080/08870440701447649 20186649

[B43] Min C. Shen F. Yu W. Chu Y. (2020). The relationship between government trust and preventive behaviors during the COVID-19 pandemic in China: exploring the roles of knowledge and negative emotion. *Prev. Med.* 141:106288. 10.1016/j.ypmed.2020.106288 33091414 PMC7571476

[B44] Montoya A. K. (2019). Moderation analysis in two-instance repeated measures designs: probing methods and multiple moderator models. *Behav. Res. Methods* 51 61–82. 10.3758/s13428-018-1088-6 30306409 PMC6420436

[B45] Oosterhoff B. Palmer C. (2020). Psychological correlates of news monitoring, social distancing, disinfecting, and hoarding behaviors among US adolescents during the COVID-19 pandemic. *PsyArXiv [Preprints].* Available Online at: 10.31234/osf.io/rpcy4. (accessed April, 2021). PMC7325067 32597925

[B46] Pearlin L. I. (1989). The sociological study of stress. *J. Health Soc. Behav.* 30 241–256. 10.2307/2136956 2674272

[B47] Pearlin L. I. Schooler C. (1978). The structure of coping. *J. Health Soc. Behav.* 19 2–21. 10.2307/2136319 649936

[B48] Piguillem F. Shi L. (2020). *Optimal COVID-19 Quarantine and Testing Policies.* CEPR Discussion Paper No. DP14613. Available Online at: https://ssrn.com/abstract=3594243. (accessed May, 2021).

[B49] Renn O. Burns W. J. Kasperson J. X. Kasperson R. E. Slovic P. (1992). The social amplification of risk: theoretical foundations and empirical applications. *J. Soc. Issues* 48 137–160. 10.1111/j.1540-4560.1992.tb01949.x

[B50] Rohmann E. Neumann E. Herner M. J. Bierhoff H. W. (2012). Grandiose and vulnerable narcissism: self-construal, attachment, and love in romantic relationships. *Eur. Psychol.* 17 279–290. 10.1027/1016-9040/a000100

[B51] Rotter J. B. (1967). A new scale for the measurement of interpersonal trust. *J. Personal.* 35 651–665. 10.1111/j.1467-6494.1967.tb01454.x 4865583

[B52] Siegrist M. Cvetkovich G. (2000). Perception of hazards: the role of social trust and knowledge. *Risk Analysis* 20 713–719. 10.1111/0272-4332.205064 11110217

[B53] Siegrist M. Gutscher H. Earle T. C. (2005). Perception of risk: the influence of general trust, and general confidence. *J. Risk Res.* 8 145–156. 10.1080/1366987032000105315

[B54] Singelis T. M. (1994). The measurement of independent and interdependent self-construals. *Pers. Soc. Psychol. Bull.* 20 580–591. 10.1177/0146167294205014

[B55] Söllner M. Dürnberger M. Keller J. Florack A. (2021). The impact of age stereotypes on well-being: strategies of selection, optimization, and compensation as mediator and regulatory focus as moderator: findings from a cross-sectional and a longitudinal study. *J. Happiness Stud.* 10.1007/s10902-021-00417-x

[B56] Thoits P. A. (1995). Stress, coping, and social support processes: Where are we? What next? *J. Health Soc. Behav.* 53–79. 7560850

[B57] Turner R. J. Avison W. R. (1992). Innovations in the measurement of life stress: crisis theory and the significance of event resolution. *J. Health Soc. Behav.* 33 36–50. 10.2307/2136856 1619257

[B58] Wang X. Cai L. Qian J. Peng J. (2014). Social support moderates stress effects on depression. *Int. J. Mental Health Syst.* 8 1–5. 10.1186/1752-4458-8-41 25422673 PMC4242489

[B59] Watson D. (1988). Intraindividual and interindividual analyses of positive and negative affect: their relation to health complaints, perceived stress, and daily activities. *J. Pers. Soc. Psychol.* 54 1020–1030. 10.1037/0022-3514.54.6.1020 3397861

[B60] Wei M. (2020). Social distancing and lockdown–an introvert’s paradise? an empirical investigation on the association between introversion and the psychological impact of COVID19-related circumstantial changes. *Front. Psychol.* 11:2440. 10.3389/fpsyg.2020.561609 PMC7527530 33041925

[B61] Wirz C. D. Xenos M. A. Brossard D. Scheufele D. Chung J. H. Massarani L. (2018). Rethinking social amplification of risk: social media and Zika in three languages. *Risk Analysis* 38 2599–2624. 10.1111/risa.13228 30408201

[B62] Worldometers (2020). *COVID-19 Coronavirus Pandemic.* Available Online at: https://www.worldometers.info/coronavirus/ (accessed June, 2020).

[B63] Ye Z. Yang X. Zeng C. Wang Y. Shen Z. Li X. (2020). Resilience, social support, and coping as mediators between COVID-19-related stressful experiences and acute stress disorder among college students in China. *Appl. Psychol. Health Well Being* 10.1111/aphw.12211 Online Head of Print. 32666713 PMC7405224

