# Supplementary material for: When Social Interaction Backfires: Frequent Social Interaction During the COVID-19 Pandemic Is Associated With Decreased Well-Being and Higher Panic Buying
Source: Front Psychol. 2021 Jul 29;12:668272. doi: 10.3389/fpsyg.2021.668272 (PMC8357996; doi:10.3389/fpsyg.2021.668272)
Supplement: Supplementary file 1 [file Data_Sheet_1.docx]

***Supplementary Materials***

**1 Study 1**

**1.1 Compliance measure**

To gauge whether our sample complied with the governmental measures, including the quarantine and social distancing rules, we included five items as an additional compliance measure. The items were produced in accordance with the general rules applied in the health sector across the European countries and the US. Participants indicated to what extent they agreed with each statement starting, “*Since the COVID-19 outbreak, I did my best*…” 1. *To cover my mouth and nose with a tissue (not with my hands) when I cough or sneeze*, 2. *To wash my hands several times a day with soap and water or a disinfectant containing alcohol*, 3. *To avoid direct contact with sick people*, 4. *To stay home as much as I can*, 5. *To keep social distance from everyone except those who live with me*, given a 7 point scale ( 1 = *strongly disagree*, 7 = *strongly agree*).

The compliance measure indicated that participants generally followed the COVID-19 measures (*M* = 6.21, *SD* = .79). As this data was extremely negatively skewed, the compliance measure was excluded from the correlational analyses. Most importantly, 90% of our sample indicated that they strongly agreed (71.6%) or agreed (18.4%) that they followed the quarantine rule (item 4), and 85.2% indicated that they strongly agreed (56.2%) or agreed (29%) that they followed the social distancing rule (item 5).

**1.2 Controlling for common method variance**

Following recommendations by Siemsen, Roth, and Oliveira (2010) and Simmering and colleagues (2015), we included a marker variable that is theoretically unrelated but acquired through the similar structure of measurements to our focal variables. We used independent self-construal as a marker variable to control common method variance by including the marker variable in the regression model we assessed. The analysis revealed that the effects of our focal variables on changes in positive and negative affects, and stress remained unchanged (see Table 1).

**Table 1 Moderated regression analyses including a marker variable in study 1**

|  | Decrease of positive affect | | | | Increase of negative affect | | | | Stress increase | | | |
| --- | --- | --- | --- | --- | --- | --- | --- | --- | --- | --- | --- | --- |
| Predictors | *b* (*se*) | 95% CI for *b* | *β* | *sr*^2^ | *b* (*se*) | 95% CI for *b* | *β* | *sr*^2^ | *b* (*se*) | 95% CI for *b* | *β* | *sr*^2^ |
| **Study 1 (*n* = 331)** |  |  |  |  |  |  |  |  |  |  |  |  |
| Social connectedness | .09 (.04) | .002, .170 | .11* | .11 | .13 (.05) | .037, .218 | .16** | .15 | .19 (.10) | .005, .380 | .12* | .11 |
| Social interaction | .08 (.02) | .039, .127 | .21** | .20 | .05 (.02) | .006, .102 | .13* | .12 | .12 (.05) | .021, .220 | .14* | .13 |
| Independent self-construal  (Marker variable) | .04 (.03) | -.024, .094 | .06 | .06 | .03 (.03) | -.029, .098 | .06 | .06 | .07 (.07) | -.060, -203 | .06 | .06 |

***p* < .01, **p* < .05

Note. *sr*^2^ indicates semi-partial (part) correlational coefficient. Changes in positive affect, negative affect, and stress are coded so that higher values indicated worsened well-being and increased stress.

**1.3 Confirmatory factor analysis of the social connectedness measure**

To show that two measures used in our studies to form a composite measure for gauging social connectedness was reasonable, we conducted a CFA which showed that a two-factor model (interdependent self-construal items and self-other overlap measure loading on factor 1; independent self-construal items loading on factor 2) showing a good model fit (*χ*^2^ = 107.41, df = 43, *p* < .001; CFI = ..852; TLI = .811; RMSEA = 0.064; SRMR = .059), better than a one-factor structure (*χ*^2^ = 232.64, df = 44, *p* < .001; CFI = .544; TLI = .429; RMSEA = 0.114; SRMR = .107), indicating that combining two measures for assessing social connectedness was justified.

**1.4 Controlling for covariates (age, gender, education, nationality)**

To verify whether any confounding variable existed, the covariates measured in Study 1 were entered as additional predictors in the tested models of the main analyses. The moderated regression analyses revealed that our main results were largely held significant while gender showed a significant effect on positive affect, indicating that females reported more decrease in positive affect than males (see Table 2).

**Table 2 Moderated regression analyses including covariates in Study 1**

|  | Decrease of positive affect | | | | Increase of negative affect | | | | Stress increase | | | |
| --- | --- | --- | --- | --- | --- | --- | --- | --- | --- | --- | --- | --- |
| Predictors | *b* (*se*) | 95% CI for *b* | *β* | *sr*^2^ | *b* (*se*) | 95% CI  for *b* | *β* | *sr*^2^ | *b* (*se*) | 95% CI  for *b* | *β* | *sr*^2^ |
| **Study 1 (*n* = 331)** |  |  |  |  |  |  |  |  |  |  |  |  |
| Social connectedness | .07 (.04) | -.010, .159 | .10 | .09 | .12 (.05) | .032, .216 | .15** | .14 | .19 (.10) | .003, .383 | .12* | .11 |
| Social interaction | .07 (.02) | .022, .111 | .17** | .15 | .04 (.03) | -.008, .090 | .10 | .09 | .12 (.06) | .002, .205 | .12* | .11 |
| Age | -.005 (.004) | -.012, .003 | -.07 | -.07 | .0001 (.004) | -.008, .008 | -.0001 | -.0001 | .002 (.009) | -.015, 020 | .02 | .02 |
| Gender  (1 = female;  2 = male) | -.17 (.07) | -.302, -.029 | -.13* | -.13 | -.15 (.08) | -.293, .004 | -.11 | -.10 | -.19 (.16) | -.492, .123 | -.07 | -.06 |
| Education | .05 (.04) | -.035, .131 | .07 | .06 | .03 (.05) | -.062, -120 | .04 | .04 | .04 (.10) | -.145, .230 | .03 | .02 |
| Nationality | .002 (.003) | -.003, .007 | .05 | .05 | .001 (.003) | -.005, .007 | .02 | .02 | .0001 (.006) | -.012, .011 | -.004 | -.004 |

**1.5 The effects of social connectedness, social interaction, and COVID-19 on *after* the pandemic measures (i.e., positive affect, negative affect, stress, general confidence level) after accounting for the effect of *before* the pandemic measures**

To control for any bias occurred in the *before* the pandemic measures, we additionally performed three hierarchical multiple regression analyses on the *after* the pandemic measures to show the effects of social connectedness and social interaction (and COVID-19 conversation for Study 2) after accounting for the effect of *before* the pandemic measures in Study 1 (Table 3-1) and Study 2 (Table 3-2). As seen in Table 3-1, the second step in the model with social connectedness and social interaction as additional predictors showed significant effects of R^2^ change, **Δ***R*^2^ = .018, **Δ***F* (2, 327) = 4.81, *p* = .001 (positive affect), **Δ***R*^2^ = .034, **Δ***F*(2, 327) = 9.64, *p* < .001 (negative affect), **Δ***R*^2^ = .051, **Δ***F*(2, 327) = 10.22, *p* < .001 (stress). The *after* the pandemic measures were strongly predicted by the *before* measures, (*β*s = .60, .62, .37, *p*s < .001), indicating that there were stable individual differences in assessments of well-being and stress. Social connectedness (*β*s = -.05, *p* = .27; .12, 11, *ps* < .01) and social interaction (*β*s > -.10, -11, .17, *p*s < .05) overall contributed significantly, indicating that participants who reported higher social connectedness and more frequent social interaction showed decreased well-being and increased stress compared to the *before* measures.

**Table 3-1 Hierarchical multiple regressions on *after* COVID-19 measures in Study 1**

|  | Positive affect *after* COVID-19 | | | | Negative affect *after* COVID-19 | | | | Stress *after* COVID-19 | | | |
| --- | --- | --- | --- | --- | --- | --- | --- | --- | --- | --- | --- | --- |
| Predictors | *b* (*se*) | *β* | *R^2^* | **Δ***R*^2^ | *b* (*se*) | *β* | *R^2^* | **Δ***R*^2^ | *b* (*se*) | *β* | *R^2^* | **Δ***R*^2^ |
| **Step 1** |  |  | .36*** |  |  |  | .39*** |  |  |  | .13*** |  |
| *before*  COVID-19 | .63  (.05) | .60*** |  |  | .65 (.05) | .62*** |  |  | .38 (.05) | .37*** |  |  |
| **Step 2** |  |  | .37*** | .02** |  |  | .42*** | .03*** |  |  | .18*** | .05*** |
| *before*  COVID-19 | .67 (.05) | .64*** |  |  | .66 (.04) | .63*** |  |  | .39 (.05) | .36*** |  |  |
| Social  Connectedness | -.049 (.04) | -.06 |  |  | .12 (.04) | .12** |  |  | .16 (.08) | .11** |  |  |
| Social interaction | -.046  (.02) | -.10* |  |  | .05 (.02) | .11* |  |  | .12 (.04) | .15* |  |  |

*** *p* < .001, ***p* < .01, **p* < .05

Likewise, as seen in Table 3-2, the second step with social connectedness, social interaction, and COVID-19 conversation as additional predictors showed significant effects of R^2^ change, **Δ***R*^2^ = .060, **Δ***F* (3, 322) = 9.54, *p* = .001 (positive affect), **Δ***R*^2^ = .13, **Δ***F* (3, 322) = 22.40, *p* < .001 (negative affect), **Δ***R*^2^ = .073, **Δ***F* (3, 322) = 14.72, *p* < .001 (general confidence). The *after* the pandemic measures were strongly predicted by the *before* measures, (*β*s = .51, .50, .63, *p*s < .001), indicating that there were stable individual differences in assessments of well-being and general confidence. Social connectedness (*β*s = -.05, *p* = .31; .12, *p* = .01; .02, *p* = .68) and COVID-19 conversation (*β*s = -.23, .31, -.23, *p*s < .001) overall contributed significantly, indicating that participants who reported higher social connectedness experienced increased negative affect and those who frequently engaged in conversations about COVID-19 topics showed decreased well-being and decreased general confidence compared to the *before* measures.

**Table 3-2 Hierarchical multiple regressions on *after* COVID-19 measures in Study 2**

|  | Positive affect *after* COVID-19 | | | | Negative affect *after* COVID-19 | | | | General confidence *after* COVID-19 | | | |
| --- | --- | --- | --- | --- | --- | --- | --- | --- | --- | --- | --- | --- |
| Predictors | *b* (*se*) | *β* | *R^2^* | **Δ***R*^2^ | *b* (*se*) | *β* | *R^2^* | **Δ***R*^2^ | *b* (*se*) | *β* | *R^2^* | **Δ***R*^2^ |
| **Step 1** |  |  | .26*** |  |  |  | .25*** |  |  |  | .39*** |  |
| *before*  COVID-19 | .56  (.05) | .51*** |  |  | .54 (.05) | .50*** |  |  | .64 (.04) | .63*** |  |  |
| **Step 2** |  |  | .32*** | .06*** |  |  | .38*** | .13*** |  |  | .47*** | .07*** |
| *before*  COVID-19 | .58 (.05) | .53*** |  |  | .53 (.05) | .49*** |  |  | .62 (.04) | .61*** |  |  |
| Social  Connectedness | -.042 (.04) | -.05 |  |  | .11 (.04) | .12* |  |  | .02 (.05) | .02 |  |  |
| Social interaction | -.007  (.03) | -.013 |  |  | .003 (.03) | .007 |  |  | -.07 (.03) | -.09 |  |  |
| COVID-19  conversation | -.146  (.03) | -.23*** |  |  | .21 (.03) | .31*** |  |  | -.20 | -.23*** |  |  |

*** *p* < .001, ***p* < .01, **p* < .05

**Table 4 Intercorrelations for measures in Study 1 including *before* and *after* measures (*n* = 331)**

| Measures | *M* (*SD*) | 1. | 2. | 3. | 4. | 5. | 6. | 7. | 8. | 9. | 10. | 11. |
| --- | --- | --- | --- | --- | --- | --- | --- | --- | --- | --- | --- | --- |
| 1. Social connectedness | 0.00 (.80) | (.56) |  |  |  |  |  |  |  |  |  |  |
| 2. Independent SC | 4.41 (1.09) | -.05 | (.68) |  |  |  |  |  |  |  |  |  |
| 3. Social  interaction | 4.95 (1.53) | .34** | -.10 | (.66) |  |  |  |  |  |  |  |  |
| 4. Information  search | 4.13 (1.31) | .17** | .04 | .35** | (.65) |  |  |  |  |  |  |  |
| 5. *before* stress | 2.65 (1.16) | -.03 | -.07 | .003 | .11^*^ | - |  |  |  |  |  |  |
| 6. *before* positive | 3.54 (.66) | .21** | .22** | .29** | .06 | -.44** | (.89) |  |  |  |  |  |
| 7. *before* negative | 2.58 (.73) | -.03 | -.12* | -.005 | .07 | .61** | -.57** | (.84) |  |  |  |  |
| 8. *after* stress | 3.11 (1.21) | .15** | -.03 | .20** | .21** | .37** | .10 | .35** | - |  |  |  |
| 9. *after* positive | 3.17 (.70) | .05 | .17** | .06 | -.04 | -.23** | .60** | -.37** | -.42** | (.88) |  |  |
| 10. *after* negative | 2.87 (.76) | .14* | -.09 | .14** | .13* | .36** | -.23** | .62** | .59** | -53** | (.84) |  |
| 11. Age | 26.95 (8.91) | -.15** | .16* | -.10 | .02 | -.09 | .03 | .001 | -.08 | .07 | -.09 | - |
| 12. Education | 2.70 (.81) | .04 | .03 | .06 | .02 | -.07 | .18** | .07 | -.002 | .09 | -.08 | .29** |

**2 STUDY 2**

**2.1 Controlling for common method variance**

As per Study 1, we used independent self-construal as a marker variable to control common method variance by including the marker variable in the regression model we assessed. The analysis revealed that the effects of our focal variables on changes in positive and negative affects, and general confidence level remained unchanged (see Table 2).

**Table 5 Moderated regression analyses including a marker variable in study 2**

|  | Decrease of positive affect | | | | Increase of negative affect | | | | General confidence decrease | | | |
| --- | --- | --- | --- | --- | --- | --- | --- | --- | --- | --- | --- | --- |
| Predictors | *b* (*se*) | 95% CI for *b* | *β* | *sr*^2^ | *b* (*se*) | 95% CI for *b* | *β* | *sr*^2^ | *b* (*se*) | 95% CI for *b* | *β* | *sr*^2^ |
| **Study 2 (*n* = 327)** |  |  |  |  |  |  |  |  |  |  |  |  |
| Social connectedness | .10 (.05) | .006 .185 | .12* | .11 | .14 (.05) | .042, .229 | .15** | .15 | -.04 (.06) | -.154, .068 | -.04 | -.04 |
| Social interaction | .04 (.03) | -.011, .095 | .09 | .08 | .03 (.03) | -.028, .082 | .05 | .05 | .07 (.03) | .003, .134 | .12* | .11 |
| COVID-19 conversation | .13 (.04) | .067, .203 | .22** | .21 | .18 (.04) | .106, .247 | .27** | .26 | .18 (.04) | .094, .262 | .24** | .22 |
| Independent self-construal  (Marker variable) | .006 (.03) | -.060, .071 | .009 | .009 | .04 (.04) | -.031, .106 | .06 | .06 | -.07 (.04) | -.155, .008 | -.09 | -.09 |

***p* < .01, **p* < .05

Note. *sr*^2^ indicates semi-partial (part) correlational coefficient. Changes in positive affect, negative affect, and general confidence level are coded so that higher values indicated worsened well-being and decreased general confidence.

**2.2 Controlling for covariates (age, gender, education, nationality)**

To verify whether any confounding variable existed, the covariates measured in Study 2 were entered as additional predictors in the tested models of the main analyses. The moderated regression analyses revealed that our main results were held significant (see Table 6). Age showed significant effects on well-being indicating that older participants reported more decreased of positive affect and more increase of negative affect. Gender also showed significant effects on the experience of positive affect and general confidence level, indicating that female participants reported more decreased of positive affect and more decrease of general confidence level compared to male participants.

**Table 6 Moderated regression analyses including covariates in Study 2**

|  | Decrease of positive affect | | | | Increase of negative affect | | | | General confidence decrease | | | |
| --- | --- | --- | --- | --- | --- | --- | --- | --- | --- | --- | --- | --- |
| Predictors | *b* (*se*) | 95% CI  for *b* | *β* | *sr*^2^ | *b* (*se*) | 95% CI for *b* | *β* | *sr*^2^ | *b* (*se*) | 95% CI for *b* | *β* | *sr*^2^ |
| **Study 2 (*n* = 327)** |  |  |  |  |  |  |  |  |  |  |  |  |
| Social connectedness | .10 (.05) | .012, .199 | .12* | .12 | .14 (.05) | .047, .233 | .16** | .15 | -.03 (.06) | -.137, .081 | -.03 | -.03 |
| Social interaction | .03 (.03) | -.024, .087 | .06 | .06 | .02 (.03) | -.038, .074 | .04 | .03 | .03 (.03) | -.037, .094 | .05 | .04 |
| COVID-19 conversation | .14 (.03) | .067, .202 | .22^***^ | 21 | .17 (.04) | .100, .241 | .26^***^ | .25 | .18 (.04) | .098, .262 | .24^***^ | .22 |
| Age | .008 (.004) | -.001, .016 | .10 | .10 | .009 (.004) | .000, .018 | .11* | .11 | .002 (.005) | -.008, 012 | .02 | .02 |
| Gender  (1 = female; 2 = male) | -.17 (.07) | -.313,  -.023 | -.12* | -.12 | -.10 (.08) | -.251, .054 | -.07 | -.07 | -.38 (.09) | -.555,-.199 | -.23^***^ | -.22 |
| Education | -.06 (.05) | -.159, .031 | -.07 | -.07 | -.01 (.05) | .090, .065 | -.01 | -.01 | .03 (.06) | -.088, .145 | .03 | .03 |
| Nationality | .002 (.004) | -.005, .008 | .02 | .02 | -.001 (.004) | -.008, .007 | -.007 | -.007 | .004 (.004) | -.004, .013 | .05 | .05 |

**Table 7-1 Intercorrelations for measures in Study 2 including *before* measures (*n* = 327)**

| Measures | *M* (*SD*) | 1. | 2. | 3. | 4. | 5. | 6. | 7. | 8. | 9. | 10. | 11. | 12. | 13. |
| --- | --- | --- | --- | --- | --- | --- | --- | --- | --- | --- | --- | --- | --- | --- |
| 1. Social connectedness | 0.00 (.81) | (.62) |  |  |  |  |  |  |  |  |  |  |  |  |
| 2. Independent SC | 4.39 (1.08) | -.02 | (.70) |  |  |  |  |  |  |  |  |  |  |  |
| 3. Information search | 4.04 (1.26) | .08 | .01 | ~~-~~ |  |  |  |  |  |  |  |  |  |  |
| 4. Social  interaction | 4.42 (1.11) | .19^**^ | .10 | .36^**^ | (.66) |  |  |  |  |  |  |  |  |  |
| 5. COVID-19  conversation | 4.42 (1.11) | .22^**^ | -.06 | .49^**^ | .31^**^ | (.81) |  |  |  |  |  |  |  |  |
| 6. Panic buying | 3.80 (1.46) | .02 | -.09 | .19^*^ | .17^**^ | .26^**^ | (.81) |  |  |  |  |  |  |  |
| 7. *before* positive | 3.62 (.65) | .19^**^ | .08 | .001 | .21^**^ | .05 | -.03 | (.88) |  |  |  |  |  |  |
| 8. *before* negative | 2.66 (.69) | -.06 | -.16^**^ | .0001 | -.09 | .08 | .04 | -.61^**^ | (.84) |  |  |  |  |  |
| 9. *before* general confidence | 3.56 (.95) | -.06 | .09 | -.06 | -.04 | -.07 | .03 | .31^**^ | -.34^**^ | (.83) |  |  |  |  |
| 10. *before* uncertainty | 3.17 (1.22) | .07 | -.06 | -.03 | -.09 | .08 | .03 | -.37^**^ | .39^**^ | -.39^**^ | - |  |  |  |
| 11. *before* fear | 2.59 (1.22) | .02 | -.08 | -.02 | -.15^**^ | .10 | .08 | -.34^**^ | .47^**^ | -.43^**^ | .63^**^ | - |  |  |
| 12. *before* anxiety | 3.08 (1.40) | -.01 | -.13^*^ | -.02 | -.11* | .10 | .03 | -.42^**^ | .52^**^ | -.34^**^ | .63^**^ | .69^**^ | - |  |
| 13. Age | 26.94 (9.02) | -.009 | .07 | -.02 | .13* | .07 | .08 | .13^*^ | -.18^**^ | .07 | -.07 | -.05 | -.11 |  |
| 14. Education | 2.61 (.78) | .002 | -.03 | -.01 | .16** | .11 | .14^*^ | .18^**^ | -.09 | .05 | -.08 | .004 | -.06 | .28** |

**Table 7-2 Intercorrelations for measures in Study 2 including *after* measures (*n* = 327)**

| Measures | *M* (*SD*) | 1. | 2. | 3. | 4. | 5. | 6. | 7. | 8. | 9. | 10. | 11. | 12. | 13. |
| --- | --- | --- | --- | --- | --- | --- | --- | --- | --- | --- | --- | --- | --- | --- |
| 1. Social connectedness | 0.00 (.81) | (.62) |  |  |  |  |  |  |  |  |  |  |  |  |
| 2. Independent SC | 4.39 (1.08) | -.02 | (.70) |  |  |  |  |  |  |  |  |  |  |  |
| 3. Information search | 4.04 (1.26) | .08 | .01 | ~~-~~ |  |  |  |  |  |  |  |  |  |  |
| 4. Social  interaction | 4.42 (1.11) | .19^**^ | .10 | .36^**^ | (.66) |  |  |  |  |  |  |  |  |  |
| 5. COVID-19  conversation | 4.42 (1.11) | .22^**^ | -.06 | .49^**^ | .31^**^ | (.81) |  |  |  |  |  |  |  |  |
| 6. Panic buying | 3.80 (1.46) | .02 | -.09 | .19^**^ | .17^**^ | .26^**^ | (.81) |  |  |  |  |  |  |  |
| 7. *after* positive | 3.06 (.71) | .001 | .07 | -.08 | .02 | -.22^**^ | -.12^*^ | (.90) |  |  |  |  |  |  |
| 8. *after* negative | 3.08 (.74) | .16^**^ | -.10 | .20^**^ | .08 | .38^**^ | .18^**^ | -.58^**^ | (.85) |  |  |  |  |  |
| 9. *after* general confidence | 2.76 (.97) | -.09 | .17^**^ | -.12^*^ | -.19^**^ | -.30^**^ | -.15^**^ | .41^**^ | -.39^**^ | (.82) |  |  |  |  |
| 10. *after* uncertainty | 4.23 (1.33) | .13^*^ | -.15^**^ | .18^**^ | .17^**^ | .40^**^ | .22^**^ | -.40^**^ | .46^**^ | -.48^**^ | - |  |  |  |
| 11. *after* fear | 3.71 (1.32) | .15^**^ | -.06 | .24^**^ | .14^**^ | .42^**^ | .27^**^ | -.43^**^ | .59^**^ | -.46^**^ | .74^**^ | - |  |  |
| 12. *after* anxiety | 4.06 (1.42) | .16^**^ | -.16^**^ | .17^**^ | .08 | .37^**^ | .24^**^ | -.47^**^ | .59^**^ | -.48^**^ | .71^**^ | .76^**^ | - |  |
| 13. Age | 26.94 (9.02) | -.009 | .07 | -.02 | .13* | .07 | .08 | -.002 | -.03 | -.02 | .07 | .14^*^ | .05 |  |
| 14. Education | 2.61 (.78) | .002 | -.03 | -.01 | .16^**^ | .11 | .14^*^ | .15^**^ | -.02 | -.04 | .10 | .12^*^ | .05 | .28^**^ |

**3 References**

Siemsen, E., Roth, A., & Oliveira, P. (2010). Common method bias in regression models with linear, quadratic, and interaction effects. *Organizational Research Methods*, *13*(3), 456-476.

Simmering, M. J., Fuller, C. M., Richardson, H. A., Ocal, Y., & Atinc, G. M. (2015). Marker variable choice, reporting, and interpretation in the detection of common method variance: A review and demonstration. *Organizational Research Methods*, *18*(3), 473-511.
